# Circulating T cell subsets are altered in individuals with chronic spinal cord injury

**DOI:** 10.1007/s12026-015-8698-1

**Published:** 2015-10-06

**Authors:** Rachel Monahan, Adam Stein, Katie Gibbs, Matthew Bank, Ona Bloom

**Affiliations:** Lab of Neuroimmunology, Feinstein Institute for Medical Research, 350 Community Drive, Manhasset, NY 11030 USA; Department of Physical Medicine and Rehabilitation, Hofstra North Shore-LIJ SOM, 1554 Northern Blvd, 4th Floor, Manhasset, NY 11030 USA; Trauma Center, Department of Surgery, North Shore University Hospital, 300 Community Drive, Manhasset, NY 11030 USA; Department of Molecular Medicine, Hofstra North Shore-LIJ SOM, Hempstead, NY 11549 USA

**Keywords:** Spinal cord injury (SCI), T cells, Regulatory T cells (T_regs_)

## Abstract

Traumatic spinal cord injury (SCI) induces changes in the immune system, both acutely and chronically. To better understand changes in the chronic phase of SCI, we performed a prospective, observational study in a research institute and Department of Physical Medicine and Rehabilitation of an academic medical center to examine immune system parameters, including peripheral immune cell populations, in individuals with chronic SCI as compared to uninjured individuals. Here, we describe the relative frequencies of T cell populations in individuals with chronic SCI as compared to uninjured individuals. We show that the frequency of CD3+ and CD3+ CD4+ T cells are decreased in individuals with chronic SCI, although activated (HLA-DR+) CD4+ T cells are elevated in chronic SCI. We also examined regulatory T cells (T_regs_), defined as CD3+ CD4+ CD25+ CD127lo and CCR4+, HLA-DR+ or CCR4+ HLA-DR+. To our knowledge, we provide the first evidence that CCR4+, HLA-DR+ or CCR4+ HLA-DR+ T_regs_ are expanded in individuals with SCI. These data support additional functional studies of T cells isolated from individuals with chronic SCI, where alterations in T cell homeostasis may contribute to immune dysfunction, such as immunity against infections or the persistence of chronic inflammation.

## Introduction

Traumatic spinal cord injury (SCI) affects approximately 276,000 Americans [1]. It is increasingly clear that many individuals living with SCI have altered immune system responses, which include hallmarks of inflammation, immunosuppression and autoimmunity [2–5]. There is also a growing appreciation of a critical balance needed between distinct innate and adaptive immune cell subsets in order to maintain normal immune function and that various aspects of this balance may be disrupted after SCI [4, 6, 7].

The mechanisms that contribute to immune dysfunction in individuals with SCI are currently unclear and likely to be multifactorial. Some aspects of immune dysfunction, particularly the high susceptibility to infection, have been partially attributed to neurogenic bowel and bladder effects. However, both the stroke and SCI clinical and research communities have noted the influence of interactions between the autonomic nervous system, particularly the sympathetic nervous system, and the immune system after neurological injury [4, 5, 8, 9]. This concept is further bolstered by recent studies of the inflammatory reflex, which describe how the vagus nerve of the parasympathetic nervous system regulates immune system function [10, 11]. For example, normal T and B cell functions are regulated by the presence of an intact vagus nerve [12, 13].

Studies in animal models demonstrated dysregulation of T cell function after SCI. Intraspinal T cells are observed later than 8 weeks post-SCI [6, 14]. In a rat model, CD3+ T lymphocytes were reduced by ~30 % for the first 2 weeks following SCI [15]. Regulatory T cells (T_regs_) have been proposed to play distinct roles within the spinal cord at different phases of recovery after SCI [16]. Depletion of CD4+ CD25+ T_regs_ improved neuronal survival after CNS injury in rats or mice, and their presence worsened neuronal survival measured at 1–2 weeks post-injury, with or without low-dose irradiation [17, 18]. The number of infiltrating T_regs_ at the injury site peaked at 2 weeks post-SCI, coinciding with their expression of the chemokine receptor CCR4 [16]. In the same study, T_regs_ played a negative role in tissue remodeling acutely and a positive role in the subacute and chronic phases of SCI, demonstrating that the roles of T cell subsets may change during phases of recovery after SCI [16].

A limited number of studies have documented altered population frequencies in vivo or activities in vitro of innate or adaptive immune cells isolated from individuals with acute or chronic SCI. In the 1990s, Campagnolo et al. [9, 19] showed that circulating lymphocytes isolated from individuals with chronic complete cervical SCI had impaired function as compared to uninjured controls; they hypothesized that this may have been due to interruptions of the sympathetic nervous system and discussed implications for infection rates in SCI. Another study of adaptive immune cells from individuals with SCI demonstrated dampened T cell function during the first 3 months post-SCI and that improvements in immune cell function correlated with rehabilitation therapy [20]. A later study by Campagnolo et al. [21] demonstrated a similar frequency of total lymphocytes, but elevated percentage of CD3+ and CD3+ CD4+ T cells in individuals with chronic SCI. Immune cell function is currently being investigated in the European SCIentinel study of “neurogenic immune depression,” with information collected within 2 and at 10 weeks after SCI, including clinical data and immune phenotypes, such as HLA-DR levels on monocytes, lymphocyte subset distribution and function ex vivo, as well as cytokines and gene expression profiling of peripheral lymphocytes [22].

Here, we characterized T cells and regulatory T cells in individuals with chronic SCI (≥1 year from initial SCI) as compared to uninjured individuals, using current molecular definitions of T cell subsets. We find a diminished frequency of total CD4+ T cells in individuals with chronic SCI, although more of them are activated. To our knowledge, we also provide the first evidence of elevated regulatory T cells in individuals with chronic SCI.

## Methods

### Study participants

The local institutional review board approved this study, and informed consent was obtained from all participants prior to study enrollment. Inclusion criteria for individuals with SCI were: ≥18 years old, a history of SCI at any level, an initial injury that occurred ≥1 year prior and injury classification with an American Spinal Injury Association Impairment Scale (AIS) grade of A–D. Potential SCI participants were excluded or study visits rescheduled if they had a concurrent infection as indicated by laboratory or clinical evidence, pressure ulcers, cancer, chemotherapy, neutropenia or autoimmune disease. Uninjured participants were ≥18 years old, without history of SCI, and selected to be within an age range similar to the chronic SCI participants. Peripheral blood samples were collected into sodium heparin-coated tubes via standard venipuncture. Data are derived from male individuals with SCI (*N* = 22 T cell, *N* = 19 T_reg_) or uninjured controls (*N* = 11, T cells and T_reg_; Table 1). Due to technical issues with sample processing or staining, one participant included in the T_reg_ analysis was not included in the T cell analysis and four participants included in the T cell analysis were not included in the T_reg_ analysis. Complete omission of these participants (*N* = 5) does not eliminate the significance (*P* values <0.05) of the findings in either the T cell or T_reg_ analysis. Additional peripheral immune cell types and immune system data analyzed from these and other participants recruited in this study will be described elsewhere.

**Table 1:** Clinical and demographic features of study participants

(A) Individual chronic SCI participants						
#	Age	Mechanism	Level	AIS	T cell	T_Reg_
A	69	Fall	T1	A	X	X
B	28	Other	T12	C	X	X
C	78	Sports	C4	D	X	X
D	45	Sports	C5	A	X	X
E	64	Fall	T8	A	X	X
F	62	Fall	C5	D		X
G	64	Sports	T2	A	X	X
H	56	MVA	C6	D	X	X
I	57	MVA	T5	A	X	X
J	58	Fall	L2	D	X	X
K	80	MVA	C8	A	X	X
L	40	MVA	C4	B	X	X
M	34	Sports	C3	C	X	X
N	63	Sports	C4	A	X	
O	60	Sports	C1	D	X	X
P	56	Fall	T7	A	X	X
Q	55	Sports	L5	D	X	
R	45	Other	C7	A	X	X
S	55	MVA	T11	B	X	X
T	52	Fall	T11	A	X	
U	72	MVA	T4	A	X	
V	59	Sports	C6	A	X	X
W	40	Other	T11	A	X	X

Individual uninjured participants			
#	Age	T cell	T_reg_
AA	55	X	X
BB	53	X	X
CC	66	X	X
DD	52	X	X
EE	53	X	X
FF	51	X	X
GG	61	X	X
HH	53	X	X
II	49	X	X
JJ	31	X	X
KK	28	X	X

(B) Summary of demographics among T cell participants			
Participants (*n*)	Uninjured	Chronic SCI	*P* value
Age (mean, SEM)	50, 3	56, 3	0.14

	Chronic SCI: *n* (%)		
Mechanism of injury		Injury level	
MVA	6 (27.3)	Cervical	10 (45.5)
Fall	5 (22.7)	Thoracic	10 (45.5)
Sport	8 (36.4)	Lumbar	2 (9.0)
Violence	3 (13.6)		
AIS grade			
A	13 (59.1)	Years from injury [mean (SEM)]	18.1 (2.7)
B	2 (9.1)		
C	2 (9.1)		
D	5 (22.7)		

(C) Summary of demographics among T_Reg_ cell participants			
Participants (*n*)	Uninjured	Chronic SCI	*P* value
Age (mean, SEM)	50, 3	56, 3	0.18

	Chronic SCI: *n* (%)		
Mechanism of injury		Injury level	
MVA	5 (26.3)	Cervical	10 (52.6)
Fall	5 (26.3)	Thoracic	8 (42.1)
Sport	6 (31.6)	Lumbar	1 (5.3)
Violence	3 (15.8)		
AIS grade			
A	10 (52.6)	Years from injury [mean (SEM)]	15.2 (2.4)
B	2 (10.5)		
C	2 (10.5)		
D	5 (26.3)		

This table provides clinical and demographic characteristics of study participants. (A) Individual participant characteristics are provided. An “X” indicates if participant was included in analysis for T cells and/or T_Reg_ panel analysis. (B) A summary of characteristics is provided for participants analyzed for each panel

### Flow cytometry

Peripheral blood leukocytes were isolated from blood using Ficoll Paque Plus Gradient (GE Healthcare), according to manufacturer’s instructions. Cells (2 × 10^6^ cells/100 μl) were incubated for 25 min on ice in the dark with antibodies conjugated to fluorophores, washed in FBS buffer (BD Biosciences Cat# 554656) and fixed in 4 % paraformaldehyde. Antibodies were purchased from BD Biosciences (BD), unless otherwise indicated. T cell panel contained the antibodies: CD3-Alexa 700 (Cat# 557943), CD4-PerCP-Cy5.5 (Cat# 560650), CD8-APC-Cy7 (Cat# 557760), CD38-APC (Cat# 555462) and HLA-DR-FITC (Miltenyi Cat# 130-095-295). (CD69 was also included in the panel, but failed to stain sufficient numbers of cells and so was not used for analysis.) The T_reg_ panel contained the following antibodies: CD3-Alexa 700 (Cat# 557943), CD4-PerCP-Cy5.5 (Cat# 560650), CD25-PE (Cat# 557138), CD127-APC (Biolegend Cat# 351316), CCR4-PE-Cy7 (Biolegend Cat# 359410) and HLA-DR-FITC (Miltenyi Cat# 130-095-295). (CD45RO was also included in the panel, but was not used in the analysis.) At least 100,000 and at most 480,000 total events were collected using a BD LSRII Flow Cytometer. Spectral compensation was performed with compensation beads (BD) on each day of staining. Analysis was performed using FlowJo software (Treestar, Inc). Cells were first gated as leukocytes using forward (FSC) versus side scatter (SSC), and then, the singlet population was selected for T cell subset analyses. Frequencies reported are of the parent populations.

### Statistical analysis

The nonparametric Mann–Whitney test was used to analyze differences between groups, with significance set at *P* < 0.05. The Kruskal–Wallis test was used to analyze differences among chronic SCI participants, according to injury levels and neurological injury status. All statistics were performed using Prism GraphPad 5 Software for Mac OSX.

## Results

### Participant characteristics

We analyzed circulating T and regulatory T cells isolated from chronic SCI (*N* = 22, 19, respectively) and uninjured (*N* = 11) participants (Table 1). Clinical and demographic features of participants are shown in Table 1. The age of uninjured participants was 50 ± 3 (mean years ± SEM) and ranged 28–66 years. The age of chronic SCI participants was 56 ± 3 (mean years ± SEM) and ranged 28–80 years. Among chronic SCI participants, the most common mechanism of injury was sports (35 %); other mechanisms included falls (26 %), motor vehicle accidents (MVAs, 26 %) or violence other (13 %). Among SCI participants, the time from initial injury ranged from 1 to 44 years; the average time from initial injury was 17 ± 2.7 (mean years ± SEM). The ASIA Impairment Scale (AIS) grades among SCI participants were: A (56.5 %), B (8.7 %), C (8.7 %) and D (26.1 %). The injury level most common among SCI participants was cervical (48 %), followed by thoracic (43 %).

### T cell characterization

**Fig. 1:**
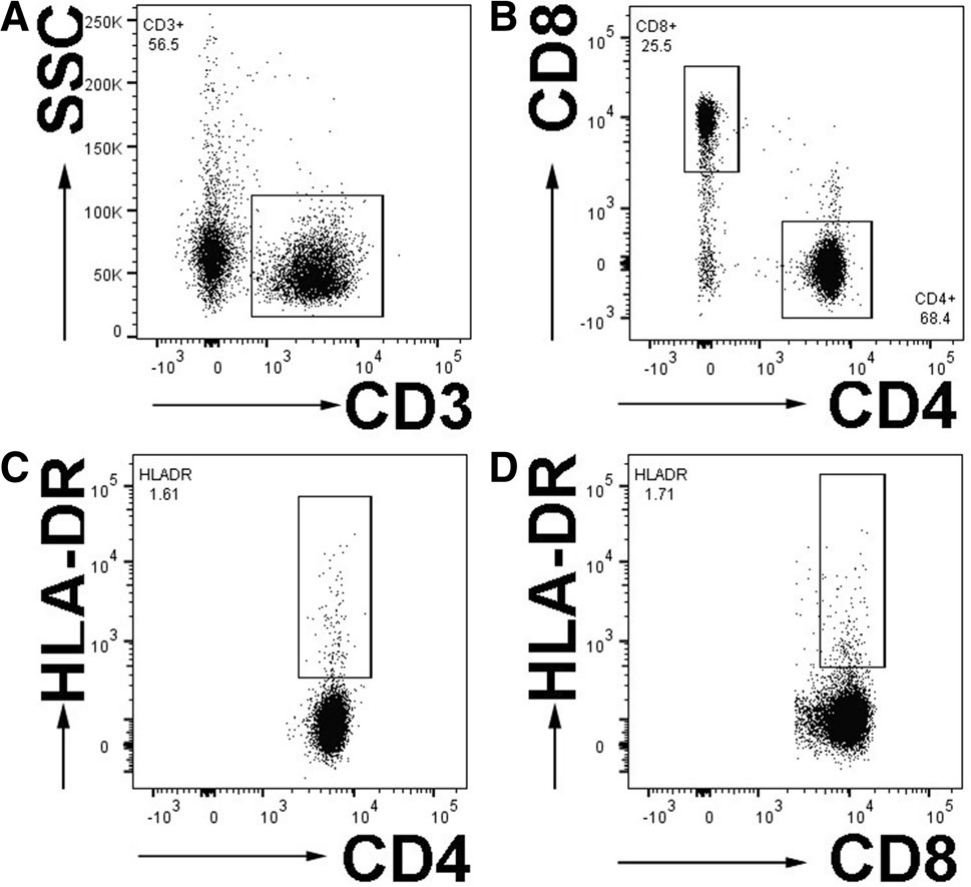
Gating strategy of T cell subsets. Flow cytometry *dot plots* illustrate the gating of all T cells, as indicated by the expression of CD3 (**a**). CD3+ cells were then gated for the expression of CD8+ or CD4+ (**b**). The proportion of activated T cells, as indicated by HLA-DR expression, was identified on CD4+ (**c**) or (**d**) CD8+ T cells

To better understand the potential roles of T cell subsets in immune dysfunction in human chronic SCI, T cell subsets were distinguished using cell surface markers currently considered to be characteristic of human T cell susbets [23]. PBMCs were isolated from blood and cell subsets labeled with multiantibody cocktails, as described in “Methods.” T lymphocyte subsets were characterized by the expression of cell surface markers: all T cells (CD3+), CD4+ or CD8+ T cells (CD3+ CD4+ or CD3+ CD8+), alone and in combination with HLA-DR (MHCII), which is present on activated human T cells [23, 24] (Fig. 1).

**Fig. 2:**
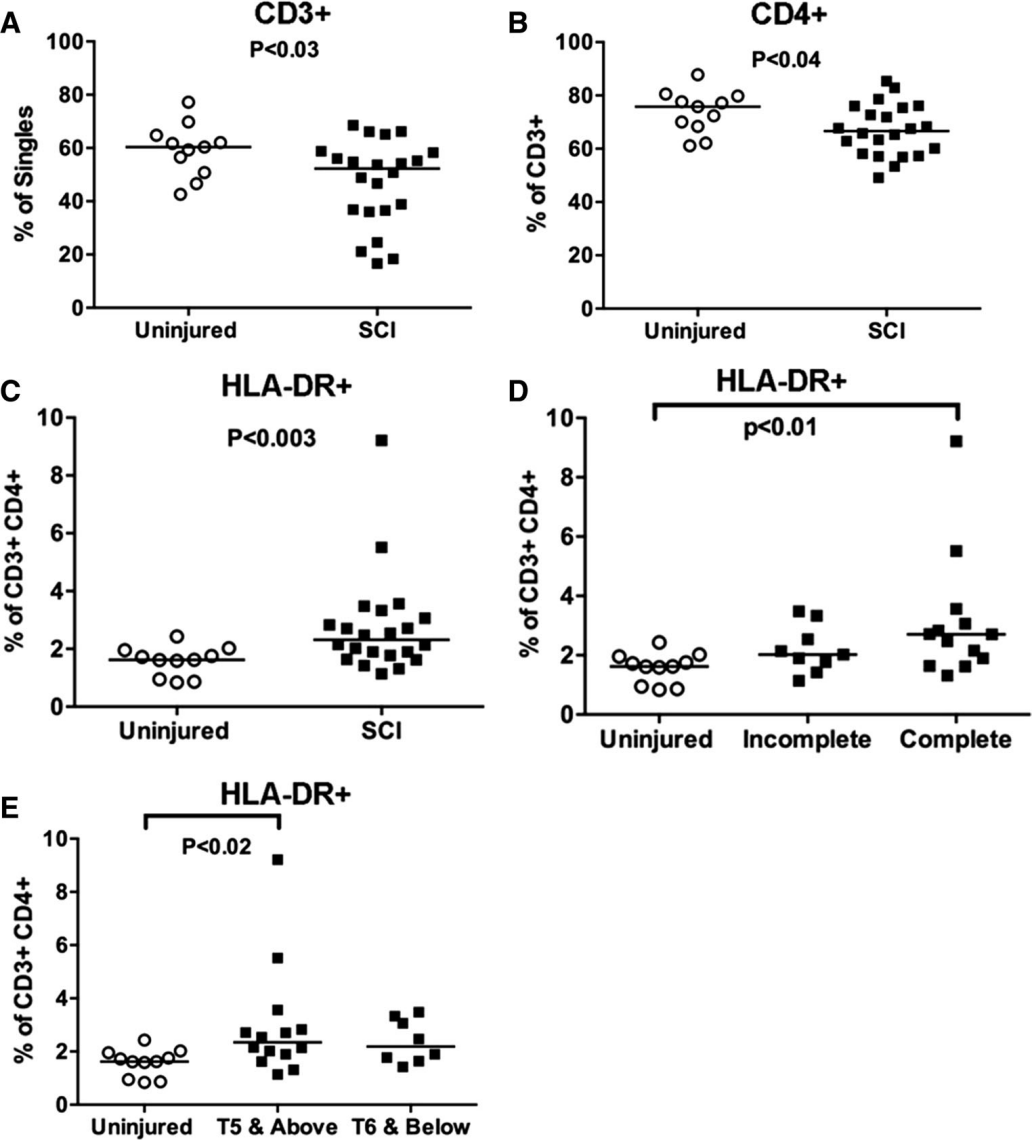
Frequency of major T cell subsets is altered in individuals with chronic SCI. Frequency of CD3+ (**a**) or CD4+ cells within the CD3+ population (**b**) is lower in individuals with chronic SCI as compared to a control group. The percentage of CD4+ cells that are activated is elevated in chronic SCI, as indicated by the expression of HLA-DR (**c**), particularly in those with neurologically complete (AIS grade A) injuries (**d**) or injuries at neurological level T5 and above (**e**). Significant *P* values are shown

Individuals with chronic SCI (*N* = 22) had a significantly lower frequency of CD3 + T cells, as compared to uninjured individuals (*N* = 11; Fig. 2a, *P* < 0.03). The percentage of CD3+ CD4+ T cells was also significantly lower in chronic SCI as compared to uninjured individuals (Fig. 2b, *P* < 0.04). However, the frequency of activated CD4+ T cells was elevated in individuals with SCI, as indicated by HLA-DR expression (Fig. 2c, *P* < 0.003), and particularly in individuals with neurologically complete injuries (*N* = 13; Fig. 2d, *P* < 0.01) or in individuals with injury levels at T5 and above (*N* = 14; Fig. 2e, *P* < 0.01). This is of interest because sympathetic nervous system innervation of immune organs occurs at T6, as described above [4, 5]. The mean fluorescence intensity of HLA-DR on CD4+ T cells was not significantly different between SCI and uninjured individuals (*P* > 0.57, mean ± SEM 937 ± 98 vs. 862 ± 76, respectively). These data indicate a disruption in homeostasis of the CD4+ T cell subset in individuals with chronic SCI.

The percentage of CD8+ T cells did not differ significantly between chronic SCI (*N* = 22) and uninjured individuals (*N* = 11; *P* < 0.19, median ± SEM median 19 ± 2 vs. 23 ± 2 %, respectively). The mean fluorescence intensity of HLA-DR on CD8+ T cells was not significantly different between SCI and uninjured individuals (*P* > 0.8, mean ± SEM 929 ± 106 vs. 1063 ± 157, respectively).

**Fig. 3:**
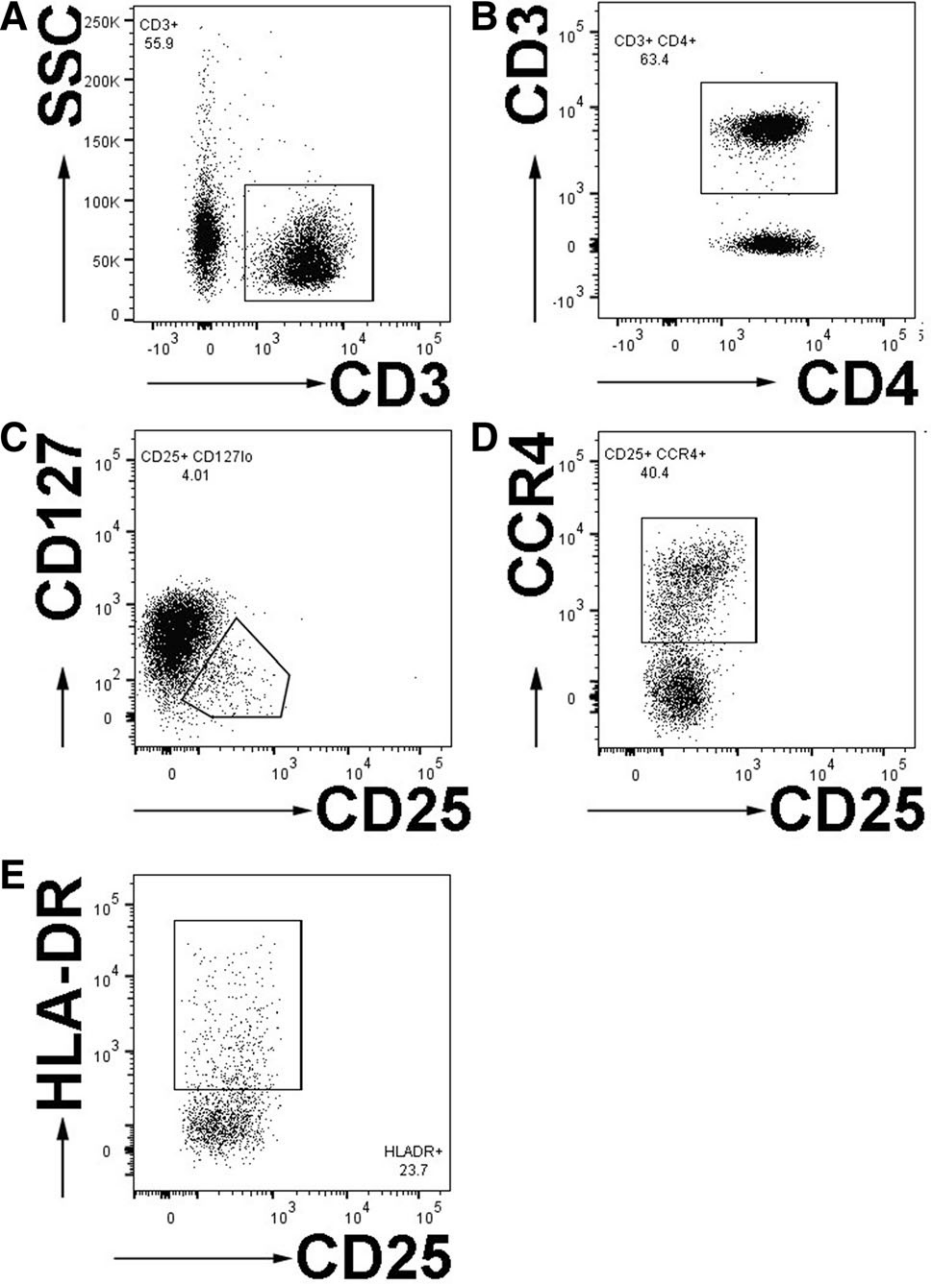
Gating strategy of regulatory CD4+ T Regs. Flow cytometry dot plots illustrate the gating of T_Regs_. Cells expressed CD3 (**a**) and CD4 (**b**). CD4+ T cells were then gated for the expression of CD25 and low expression of CD127 (**c**). CD25+ CD127lo T cells were then gated for the expression of CCR4 (**d**) and HLA-DR (**e**)

Regulatory T cells are a heterogeneous population of T cells which can suppress activation of most other innate and adaptive immune cell types and are dysregulated in neurological and non-neurological chronic diseases, including multiple sclerosis, type I diabetes and rheumatoid arthritis [7, 25, 26]. Due to their importance in modulating immune responses generally and reports of immune depression in SCI [4, 5, 22, 27, 28], we also measured the frequency of regulatory T cells (T_Regs_) here. While the transcription factor FoxP3 is the most widely used marker for murine CD4+ T_Regs_, it requires intracellular staining (which precludes functional assays) and its expression patterns and stability in humans are less well understood [7]. CD4+ T_Regs_ express CD3+ CD4+ and high levels of CD25, the IL-2 receptor. The chemokine receptor CCR4+, which facilitates migration, was also shown to be present on most human peripheral blood T_Regs_, where it correlates with the expression of Foxp3+ [23, 29, 30]. CD127, the alpha chain of the IL-7 receptor, is inversely correlated with FoxP3+ in human T_Regs_ [31, 32] and in combination with CD25+ high CD4+ T cells, identifies T_Regs_ in human peripheral blood [33, 34]. Expression of HLA-DR on T_Regs_ is currently accepted to define a population of terminally differentiated, effector T_Regs_ with high suppressive activity [25, 35]. This population is functionally defective in cells isolated from individuals with multiple sclerosis [25]. Therefore, to characterize T_Regs_ here, we used a gating strategy that included the expression of CD3+, CD4+, high expression of CD25, low or no expression of CD127, and expression of CCR4, HLA-DR (MHCII) or both CCR4 and HLA-DR (Fig. 3).

**Fig. 4:**
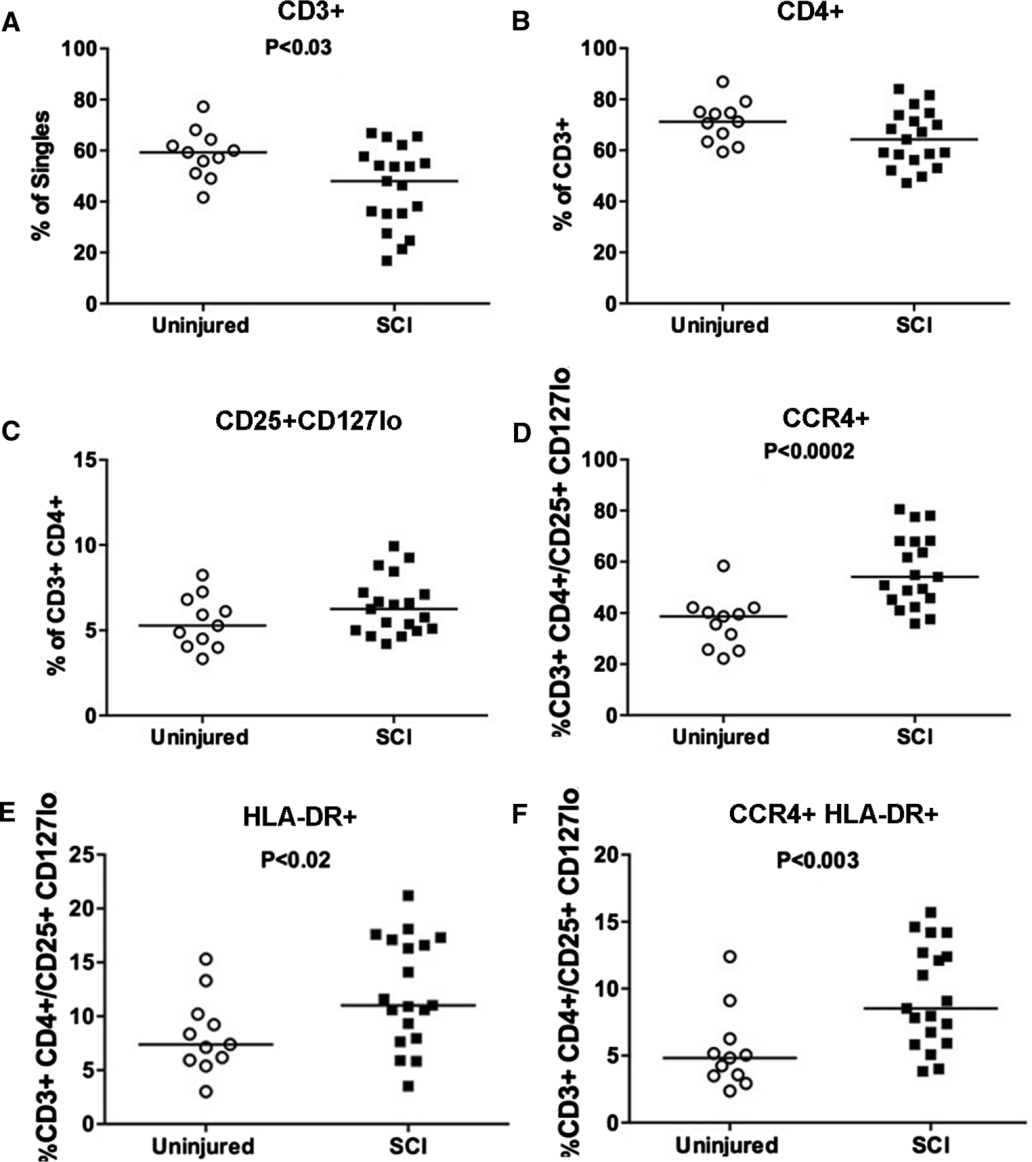
Frequency of T_Reg_ populations is altered in individuals with chronic SCI. The frequency of CD3+ (**a**) is again lower in this group of individuals with chronic SCI as compared to a control group, while a trend toward a lower CD4+ cell population (**b**) is observed in individuals with chronic SCI. The frequency of CD25+ CD127lo expressing cells is equivalent between uninjured and individuals with chronic SCI (**c**). Within the CD25+ CD127lo population, the frequencies of CCR4+ or HLA-DR+ cells are significantly elevated in individuals with chronic SCI (**d**, **e**). Within the CD25+ CD127lo population, the frequency of cells that were positive for CCR4 and HLA-DR was also elevated (**f**). Significant *P* values are shown

Using the T_Regs_ antibody panel, we again observed a lower percentage of CD3+ T cells (*P* < 0.03, Fig. 4a) and a trend toward a lower percentage of CD4+ T cells in individuals with chronic SCI (*N* = 19) as compared to uninjured individuals (*N* = 11; *P* < 0.07, Fig. 4b). The percentage of CD3+ CD4+ T cells that were CD25+ CD127lo was not significantly different between chronic SCI and uninjured individuals (*P* < 0.15, Fig. 4c). However, we observed a significant increase in the proportion of CD25+ CD127lo CD4+ T cells that expressed CCR4+ (*P* < 0.0002, Fig. 4d). Among individuals with SCI, this difference was significant in individuals with neurologically incomplete (*N* = 9) or complete injuries (*N* = 10) (*P* < 0.01, *P* < 0.05, respectively) and in individuals with injuries at level T5 and above (*N* = 13) or T6 and below (*N* = 6; *P* < 0.01 and *P* < 0.05, respectively). We also observed a significant increase in the proportion of CD25+ CD127lo CD4+ T cells that expressed HLA-DR (*P* < 0.02, Fig. 4e) or both CCR4+ and HLA-DR+ (*P* < 0.003, Fig. 4f).

## Discussion

Here, we find disruptions in the frequencies and activation status of different subsets of peripheral T cells in individuals with chronic SCI. Specifically, we find decreased frequencies of CD3+ and CD4+ T cells, particularly in individuals with complete or high level SCI (Figs. 2a, b, 4a, b). However, the frequency of activated CD4+ T cells was elevated in individuals with SCI (Fig. 2c–e). We also find elevated frequencies of CD4+ T_regs_ in individuals with chronic SCI, particularly in CD25+ CD127loCCR4+ (Fig. 4d), CD25+ CD127loHLA-DR+ (Fig. 4e) and CD25+ CD127loCCR4+ HLA-DR+ (Fig. 4f) populations.

T cells are critical for proper function of the adaptive immune system and have been studied in the context of responses to infection in SCI models. After mouse hepatitis virus infection 1 week post-injury, SCI mice had increased mortality compared to infected mice without SCI, which was accompanied by reduced IFNy+ CD4+ T cells, virus-specific T cells and total CD4+ T cells, in addition to increased viral replication [36]. In another study of chronic SCI in mice, CD4+ and CD8+ T cell function were also decreased, as indicated by diminished cytokine production ex vivo and higher expression of an exhaustion markers [37]. Interestingly, the same study showed that CD8+ dysfunction was promoted by impairment of the sympathetic nervous system and could be modulated by norepinephrine [37].

Much less is known about T cell biology in human SCI, but there is evidence of altered T cell biology. CD4+ T cell frequency and function are diminished in individuals with acute SCI [8, 28]. There is also evidence of altered CD4+ T cell biology in later phases of recovery after SCI. A study of SCI cadavers showed that elevated numbers of intraspinal CD4+ and CD8+ T cells were present weeks after SCI [38]. T cells isolated from bone marrow of individuals with chronic SCI had impaired activity in vitro [39]. Serum IL-2R (CD25) levels and lymphocyte proliferation decreased in individuals within the first 3 months post-SCI and then increased to normal or near-normal levels at year post-injury [8, 20, 40]. Infections were recently identified as an independent risk factor for poor neurological recovery for the first year after SCI and are also a risk factor for poor neurological recovery in stroke [8, 27, 41, 42]. Taken together, the present findings of altered T cell subsets in individuals chronic SCI may be relevant to infection susceptibility among this population as well as to other manifestations of immunosuppression or immune system dysfunction observed in chronic SCI [4, 5]. Additional studies are necessary to evaluate the relevance of these findings to the general SCI population and to investigate whether functional activities of altered T cell subsets, such as helper and suppressor activities, are also altered in cells isolated from individuals with SCI.

Study limitations include a relatively small cohort size and unequal gender distribution, so only data from male participants are described. Among SCI participants, the time from initial injury varied greatly, we did not recruit an equal number of individuals with neurologically complete or incomplete injuries, nor did we recruit an equal number of individuals with high or low injury levels. Also, we did not collect information on acute SCI in the same individuals, nor did we collect information on participants’ rehabilitation programs or general activity levels, which may potentially influence immune responses [20, 40].
